# Antibacterial and Antibiofilm Effects of Lactobacilli Strains against Clinical Isolates of *Pseudomonas aeruginosa* under Conditions Relevant to Cystic Fibrosis

**DOI:** 10.3390/antibiotics12071158

**Published:** 2023-07-07

**Authors:** Giovanna Batoni, Elisa Catelli, Esingül Kaya, Arianna Pompilio, Marta Bianchi, Emilia Ghelardi, Giovanni Di Bonaventura, Semih Esin, Giuseppantonio Maisetta

**Affiliations:** 1 Department of Translational Research and New Technologies in Medicine and Surgery, University of Pisa, Via S. Zeno 37, 56123 Pisa, Italy; e.catelli@studenti.unipi.it (E.C.); e.kaya@studenti.unipi.it (E.K.); marta.bianchi@phd.unipi.it (M.B.); emilia.ghelardi@unipi.it (E.G.); semih.esin@unipi.it (S.E.); 2 Department of Medical, Oral, and Biotechnological Sciences, G. d’Annunzio University of Chieti-Pescara, Via dei Vestini, 31, 66100 Chieti, Italy; arianna.pompilio@unich.it (A.P.); gdibonaventura@unich.it (G.D.B.); 3 Center for Advanced Studies and Technology, G. d’Annunzio University of Chieti-Pescara, Via L. Polacchi 11, 66100 Chieti, Italy

**Keywords:** lactobacilli, *Pseudomonas aeruginosa*, cystic fibrosis, artificial sputum medium, biofilm, tobramycin

## Abstract

Therapy of lung infections sustained by *Pseudomonas aeruginosa* in cystic fibrosis (CF) patients is challenging due to the presence of a sticky mucus in the airways and the ability of the bacterium to form biofilm, which exhibits increased antibiotic tolerance. A lung-directed bacteriotherapy through the airway administration of probiotics could represent an alternative approach to probiotic diet supplementation to improve the benefits and clinical outcomes of this kind of intervention in CF patients. This study aims to evaluate the ability of probiotic strains to grow in artificial sputum medium (ASM), mimicking the CF lung microenvironment, and to affect the planktonic and biofilm growth of CF clinical strains of *P. aeruginosa* in the same conditions. The results demonstrate that *Lacticaseibacillus rhamnosus* and *Lactiplantibacillus plantarum* (LP) can grow in ASM. LP inhibited the planktonic growth of *P. aeruginosa*, while both lactobacilli reduced the pre-formed biofilm of *P. aeruginosa*. Interestingly, LP was demonstrated to reduce the amount of polysaccharides in the extracellular matrix of *P. aeruginosa* biofilms and to potentiate the antibiofilm effects of tobramycin. Overall, the results indicated that LP is a promising candidate as an adjuvant in the antimicrobial therapy of *P. aeruginosa* infections in CF patients.

## 1. Introduction

Cystic fibrosis (CF) is a genetic disorder characterized by impaired chloride ion channel function, which results in thick mucus secretions in the lungs and increased susceptibility to chronic bacterial infections [1]. *Staphylococcus aureus* and *Pseudomonas aeruginosa* are among the most prevalent pathogens chronically colonizing CF airways, with the former mainly infecting the patients in infancy or early childhood and the latter prevailing in adolescence and early adulthood, where it is associated with a worse clinical course [2]. While the early infecting *P. aeruginosa* strains impair *S. aureus* growth due to competitive interactions, in the later stages of chronic infections, cooperative interactions between *S. aureus* and *P. aeruginosa* have been described, promoting the co-existence of the two bacterial species in a proportion of patients [3,4,5]. Although poly-microbial infections are gaining growing interest in CF patients, *P. aeruginosa* remains a major pathogen in such patients. The therapy of *P. aeruginosa* infections is indeed particularly challenging due to the intrinsic resistance of the bacterium to many antibiotics and its ability to readily evolve resistance to new antibiotics [6]. Another major challenge associated with the treatment of pulmonary *P. aeruginosa* infections in CF patients is the ability of the bacterium to develop biofilm that protects the microorganism from antibiotics and host immunity [7].

Despite the advent of modulator therapies for the Cystic Fibrosis Transmembrane Regulator (CFTR), respiratory infections by *P. aeruginosa* remain a major issue in CF patients, keeping the interest in identifying new antimicrobial strategies to substitute or complement antibiotic use high [8]. Recent studies report a tendency of the lung microbiome to increment in richness and diversity following CFTR modulator therapy [8,9]. However, a reduction in pulmonary *P. aeruginosa* load is reported in some but not all of the studies [10]. It is also noteworthy that although in several studies, ivacaftor therapy has been demonstrated to reduce *P. aeruginosa* load in sputum, it rarely eradicates the bacterium that has instead been reported to later rebound in most of the samples [11,12].

In this context, probiotic bacteria, either as prophylactic agents for preventing or delaying pulmonary colonization with CF pathogens or eventually as therapeutic tools to fight established pulmonary infections, represent a recently emerged and particularly attractive strategy [13]. Probiotics—‘live microorganisms which, when administered in adequate amounts, confer a health benefit on the host’—have been traditionally exploited, with undiscussed efficacy, to treat human intestinal disorders [14,15]. Nevertheless, evidence is accumulating in favor of the use of probiotics in many extra-intestinal clinical applications, including respiratory tract infections. For instance, randomized controlled trials have demonstrated that probiotic supplementation reduces pulmonary exacerbations and improves the quality of life of children with CF [16,17,18]. A recent systematic review addressing the use of probiotics in CF patients indicates a promising future for this type of intervention [19]. Although the mechanisms by which orally administered probiotic strains exert their beneficial effects in human respiratory infections are still unclear, they likely rely on the gut–lung axis, i.e., a complex inter-organ crosstalk where the gut microbiota may positively affect distant sites like the lung.

Rationally, the therapeutic effect of probiotics might be potentiated by directly administering them into the respiratory tract (via aerosol or intranasal inoculation), as this route would allow a direct effect of probiotics against the lung pathogens, meanwhile modulating the local immune responses. Supporting evidence for this hypothesis is accumulating. Recent reports have demonstrated that intra-tracheal and intra-nasal administrations of lactobacilli in mice protect the animals from *P. aeruginosa* pulmonary infection [20,21]. Notably, a high prevalence of lactobacilli carriage was observed in the lungs of CF patients [22]. Three species of lactobacilli *(Lacticaseibacillus rhamnosus*, *Lacticaseibacillus paracasei*, and *Lactobacillus gasseri*) found in the CF lungs are reported to be the most prevalent in the oral cavity and are known to be part of the gastrointestinal tract. This suggests that oral or intestinal lactobacilli could transiently colonize CF patient lungs through gastroesophageal reflux and/or micro-breathing [22].

Some lactobacilli, such as *Lactiplantibacillus plantarum* (LP) and *Lacticaseibacillus rhamnosus* (LR), have been shown to impair the growth of *P. aeruginosa* in vitro [23,24]. However, their ability to survive and/or exert their antibacterial effect in CF-like conditions has not been previously investigated.

In view of the potential use of lactobacilli by respiratory route administration in CF patients, the main aim of this study was to evaluate the survival ability and the antimicrobial/anti-biofilm potential of commercial lactobacilli strains against clinical isolates of *P. aeruginosa* in an artificial sputum medium (ASM) that resembles CF lung fluid. 

## 2. Results

### 2.1. Ability of Probiotic Strains to Grow and/or Survive in Artificial Sputum Medium (ASM)

**Figure 1:**
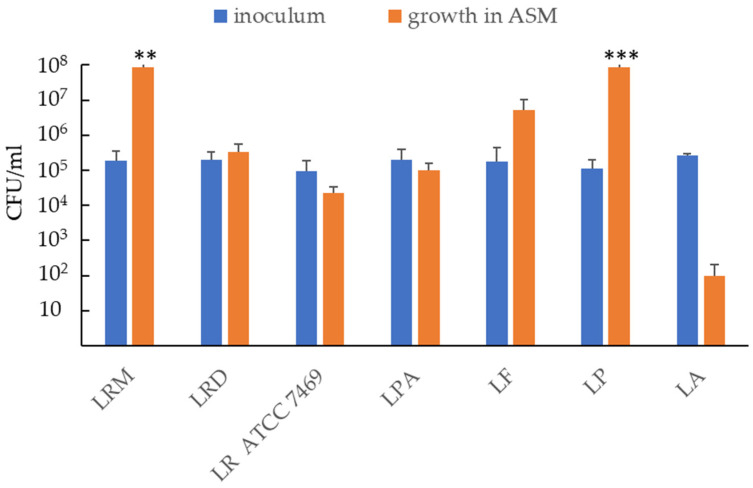
Ability of probiotic strains to grow/survive in artificial sputum medium (ASM). The figure reports the CFU number of lactobacilli strains after 24 h of incubation in ASM as compared to the initial inoculum. Results are shown as mean ± standard error of the mean values (*n* = 5). Statistical significance was evaluated by Student’s *t*-test. ** *p* < 0.01; *** *p* < 0.001.

To mimic the CF lung environment, we used a previously described formulation of ASM [25] with modifications to facilitate lactobacilli growth. In particular, we supplemented ASM with glucose at the mean concentration measured in the sputum of CF patients [26], as glucose is essential for lactobacilli survival (Figure S1). We tested the ability of several species/strains of commercial lactobacilli to survive/grow in the modified ASM. The strain code used throughout the study and the commercial sources are shown in Table S1. To this end, 10^5^ CFU/mL of each strain/species of lactobacilli was inoculated in ASM. After 24 h of incubation at 37 °C under microaerophilic conditions, serial dilutions of the bacterial suspensions were plated on a solid medium, and the number of CFU/mL was compared with that of the initial inoculum. As reported in Figure 1, different lactobacilli showed variable abilities to survive/grow in ASM. *Lacticaseibacillus rhamnosus* (Microbiosys, LRM), *Lactiplantibacillus plantarum* (LP), and, to a lesser extent, *Limosilactobacillus fermentum* (LF) showed a marked increase in CFU number as compared to the inoculum. However, statistical significance was only reached in the cases of LRM and LP. Contrarily, the numbers of *L. rhamnosus* (Dicoflor, LRD), *L. rhamnosus* ATCC 7469 (LR ATCC 7469), and *Lacticaseibacillus paracasei* (LPA) remained stable over 24 h, whereas *L. acidophilus* (LA) showed poor survival in ASM.

### 2.2. Anti-Bacterial Effect of Lactobacilli on Planktonic P. aeruginosa in ASM

**Figure 2:**
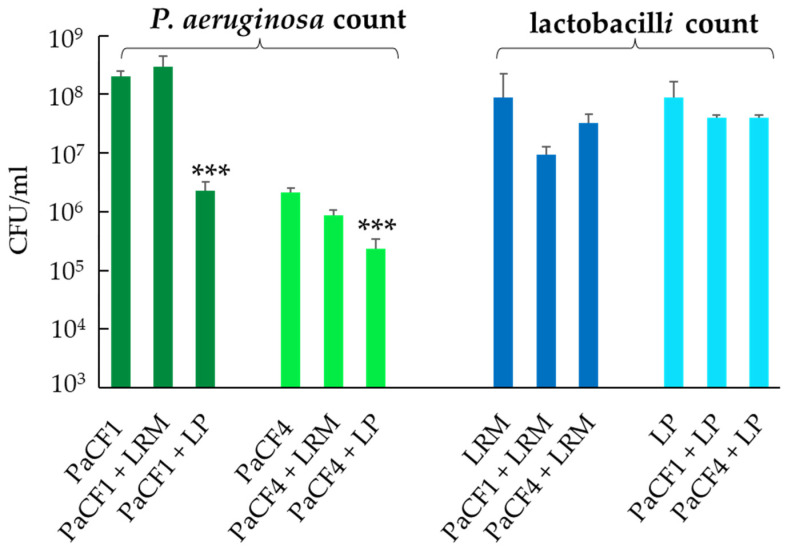
Antibacterial effect of lactobacilli on planktonic *P. aeruginosa* in ASM. *L. plantarum* (LP) and *L. rhamnosus* (LRM) (10^5^ CFU/mL) were grown in ASM for 9 h, before the addition of 10^3^ CFU/mL of *P. aeruginosa* strains (PaCF1 or PaCF4). After an additional 13 h of incubation, bacterial suspensions were serially diluted and plated on MRSA and MacConkey for enumeration of lactobacilli and *P. aeruginosa,* respectively. Results are shown as mean ± standard error of the mean values (*n* = 4). Statistical significance was evaluated by ANOVA followed by the Tukey–Kramer posthoc test. *** *p* < 0.001.

Based on the growth data in ASM, LP and LRM were chosen to evaluate their antibacterial activities against two clinical strains of *P. aeruginosa* isolated from the sputum of chronically infected CF patients, namely PaCF1 (non-mucoid) and PaCF4 (mucoid) (Table S2). To this end, each strain of lactobacilli was grown in ASM for 9 h before adding *P. aeruginosa*, and thereafter, the co-incubation was prolonged for an additional 13 h. As shown in Figure 2, a statistically significant reduction in CFU of both PaCF1 and PaCF4 was observed in the samples pre-incubated with LP as compared to the controls containing *P. aeruginosa* alone. In contrast, in the adopted conditions, no evident reduction in the bacterial load of both *P. aeruginosa* strains was observed when the samples were pre-incubated with LRM (Figure 2). 

pH measurement of ASM following co-culture of PaCF1 and PaCF4 with LP and LRM indicated a marked lowering of the pH values (below 6) in the presence of LP and, to a lesser extent, in the presence of LRM (Figure S2). This evidence may suggest that the acidity generated in the medium by LP may contribute to the inhibitory effect on *P. aeruginosa*.

### 2.3. Coaggregation Ability of LP and LRM vs. P. Aeruginosa

**Figure 3:**
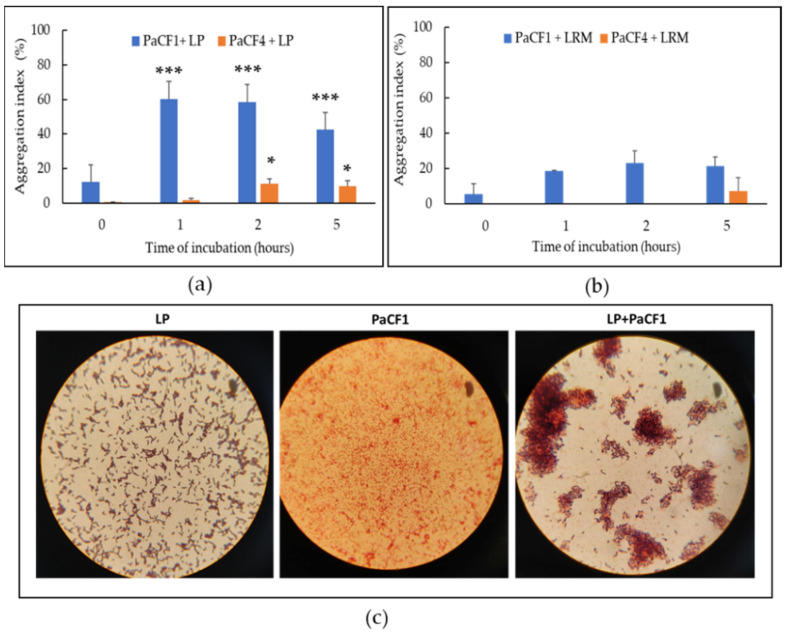
Ability of LP (**a**) and LRM (**b**) to coaggregate with PaCF1 and PaCF4 at different times of incubation in PBS (pH 7.4). (**c**) Gram staining of monocultures and co-cultures of LP and PaCF1. Results are shown as mean ± standard error of the mean values (*n* = 5). Statistical significance was evaluated by ANOVA followed by the Tukey–Kramer posthoc test. * *p* < 0.05, *** *p* < 0.001.

Coaggregation is a recognized mechanism through which lactobacilli can exert their probiotic effects by creating a hostile microenvironment around pathogens and preventing their adhesion to surfaces [27]. Therefore, we tested the ability of lactobacilli to bind *P. aeruginosa* by performing a coaggregation assay in PBS at different co-incubation times. As shown in Figure 3**,** LP displayed a marked coaggregation ability toward PaCF1 (60%) and to a lesser extent toward PaCF4 (15%) (Figure 3a). In contrast, LRM displayed a poor ability to coaggregate with both strains (Figure 3b). The ability of LP to coaggregate with PaCF1 was also visualized by Gram staining of monocultures and co-cultures of LP and PaCF1 (Figure 3c).

### 2.4. Effects of Lactobacilli on Biofilm Formation and Pre-Formed Biofilms of P. aeruginosa in ASM

**Figure 4:**
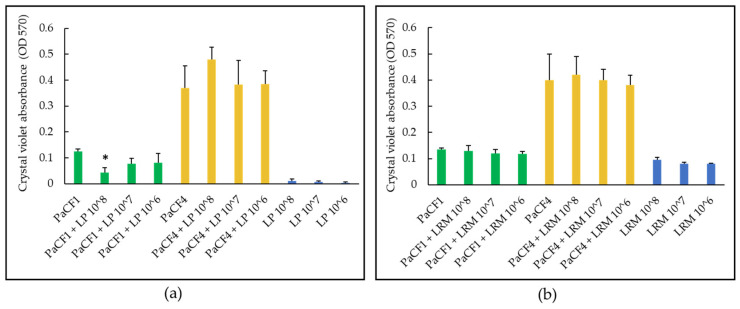
Effects of different concentrations (10^6^, 10^7^, 10^8^ CFU/mL) of LP (**a**) and LRM (**b**) on the biofilm biomass formed by PaCF1 (green bars) and PaCF4 (yellow bars) strains in ASM, as assessed by the crystal violet assay. Results are shown as mean ± standard error of the mean values (*n* = 3). Statistical significance was evaluated by ANOVA followed by the Tukey–Kramer posthoc test. * *p* < 0.05.

We next assessed the capacity of LP and LRM to inhibit the biofilm formation by *P. aeruginosa* strains PaCF1 and PaCF4. The antibiofilm effect of both LP and LRM tested at different concentrations (10^6^, 10^7^, 10^8^ CFU/mL) against *P. aeruginosa* was assessed in ASM and evaluated by crystal violet staining (total biofilm biomass) and by CFU count (biofilm-associated viable *P. aeruginosa*). As shown in Figure 4a, LP inhibited the formation of the biofilm of PaCF1 (but not of PaCF4), reducing the total biofilm biomass by more than 50% as compared to the untreated sample at the highest concentration tested (10^8^ CFU/mL). The biofilm formation of both PaCF1 and PaCF4 was not affected by LRM (Figure 4b).

Biofilm-associated viable count of both PaCF1 and PaCF4 co-incubated with either LP or LRM was not reduced compared to the control (*P. aeruginosa* alone) (Figure S3a). 

To investigate the effects of LP and LRM on pre-formed biofilm, different doses of lactobacilli (10^6^, 10^7^, 10^8^ CFU/mL) were incubated with 24-h-old mature biofilms of three *P. aeruginosa* strains: one non-mucoid (PaCF1) and two mucoid (PaCF4 and PaCF11). After an incubation of 24 h in ASM, biofilm biomass and biofilm-associated viable cell numbers were evaluated.

**Figure 5:**
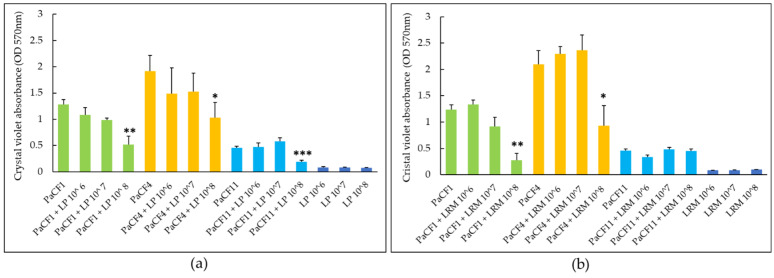
Effects of different concentrations (10^6^, 10^7^, 10^8^ CFU/mL) of LP (**a**) and LRM (**b**) on preformed biofilm of PaCF1 (green bars), PaCF4 (yellow bars) and PaCF11 (light blue bars) strains in ASM, as assessed by the crystal violet assay. Results are shown as mean ± standard error of the mean values (*n* = 4). Statistical significance was evaluated by ANOVA followed by the Tukey–Kramer posthoc test. * *p* < 0.05, ** *p* < 0.01, *** *p* < 0.001.

LP caused a statistically significant reduction of the biofilm biomass of all three *P. aeruginosa* strains when tested at 10^8^ CFU/mL (Figure 5a). At the same concentration, LRM caused a statistically significant reduction of the biofilm biomass of PaCF1 and PaCF4, but not of PaCF11 (Figure 5b). In the case of LP, the biomass reduction of PaCF1, PaCF4, and PaCF11 was 57%, 46%, and 60%, respectively, compared to the untreated samples (Figure 5a), while in the case of LRM, such reduction was 77% and 56% towards PaCF1 and PaCF4, respectively (Figure 5b). When incubated alone, both strains of lactobacilli did not form an appreciable biofilm in ASM (Figure 5a,b). 

Despite the reduction in biofilm biomass, no evident reduction in biofilm-associated viable count was observed when both lactobacilli were incubated with pre-formed biofilms of each *P. aeruginosa* strain (Figure S3b).

### 2.5. Confocal Microscopy Analysis of P. aeruginosa Biofilm after Exposure to LP

**Figure 6:**
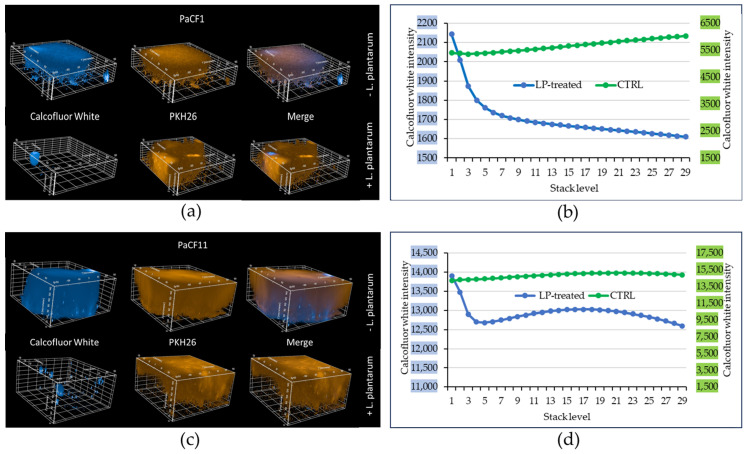
Analysis of the effect of LP on the extracellular matrix of the pre-formed biofilm of *P. aeruginosa*. Three-dimensional reconstruction of the biofilm of *P. aeruginosa* PaCF1 (non-mucoid strain) (**a**) and PaCF11 (mucoid strain) (**c**) after labeling with Calcofluor white and PKH26. Intensity of Calcofluor white at different Z stacks (biofilm layers from the most superficial, 29, to the deepest 1, for PaCF1 (**b**) and PaCF11 (**d**) strains. CTRL: *P. aeruginosa* not exposed to LP. Data from a representative experiment.

The significant reduction in biofilm biomass of preformed biofilms along with no appreciable reduction in CFU count suggested that the anti-biofilm effect of lactobacilli could be attributable to a decrease in the biofilm extracellular polymeric substance (EPS). To verify this hypothesis, the biofilm EPS was marked using the Calcofluor white fluorescent tracer, a non-specific dye capable of staining the β-polysaccharides which are very abundant in the *P. aeruginosa* matrix [28]. Bacterial cells were labeled, instead, with the lipophilic dye PHK26 that binds to bacterial cell membranes. Pre-formed biofilms of PaCF1 and PaCF11 were treated with LP (10^8^ CFU/mL) for 24 h before labeling the biofilms with the two fluorescent dyes and visualizing them via confocal microscopy. As shown in the 3D reconstruction of the biofilm of PaCF1 (Figure 6a) and PaCF11 (Figure 6c), a marked reduction in the amount of EPS (Calcofluor White, blue color) was visible in the samples treated with LP compared to the untreated control, while no evident difference in PKH26 staining (orange color) was evident. The intensity of Calcoflour white in the different Z stacks (optical sections of the biofilm) was also quantified using a dedicated software. The untreated biofilms did not show variations of fluorophore intensity in all stack levels (Figure 6b,d). In contrast, biofilms treated with LP showed a lower intensity of the fluorophore than the control sample in stack 29 (the most superficial layer of the biofilm) and through the thickness of the biofilm down to stack 5, while the intensity progressively raised in the deeper layers of the biofilm (stacks 4, 3, 2 and 1). Such a trend was evident for both strains of *P. aeruginosa* (Figure 6b,d).

### 2.6. Activity of Tobramycin against Pre-Formed Biofilms of P. aeruginosa (PaCF1 and PaCF11 strains) Pre-Treated with LP

We hypothesized that the ability of LP to reduce *P. aeruginosa* biofilm extracellular matrix could favor the penetration of antibiotics into the biofilm and, therefore, enforce their activity. Since tobramycin is currently used as a front-line drug for treating CF lung infections, lactobacilli + tobramycin synergism experiments were carried out. We first evaluated the MIC values of tobramycin towards both *P. aeruginosa* strains (PaCF1 and PaCF11), and LP. MIC values of tobramycin against LP (25 μg/mL) were much higher than those obtained against *P. aeruginosa* strains (0.25 and 1 μg/mL, for PaCF1 and PaCF11, respectively), suggesting that the concentrations of tobramycin active against *P. aeruginosa* would not inhibit LP. 

**Figure 7:**
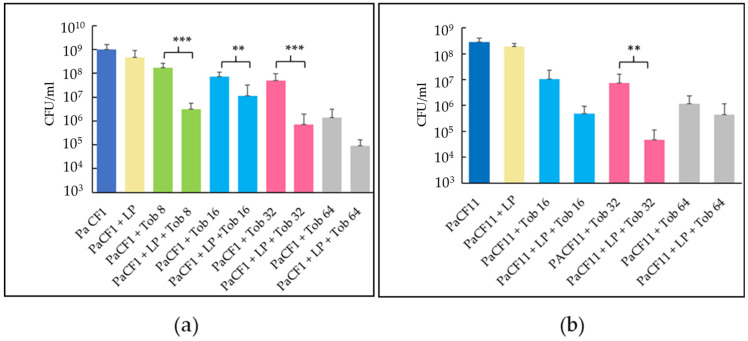
Activity of tobramycin (Tob) at different concentrations on preformed biofilm of PaCF1 strain (**a**) and PaCF11 strain (**b**) pretreated or not with LP. The concentrations of tobramycin are expressed in μg/mL. Results are shown as mean ± standard error of the mean values (*n* = 3). Statistical significance was evaluated by ANOVA followed by the Tukey–Kramer posthoc test, ** *p* < 0.01, *** *p* < 0.001.

Subsequently, we treated 24-h-old biofilms of PaCF1 and PaCF11 obtained in ASM with sequential exposure to LP (10^8^ CFU/mL) for 24 h and tobramycin for a further 24 h. Pre-exposure of PaCF1 to LP followed by treatment with tobramycin at 8, 16, or 32 μg/mL caused a statistically significant decrease of PaCF1 CFU as compared to the control treated with the antibiotic alone (Figure 7a). Similar results were obtained with the PaCF11 strain using tobramycin at 32 μg/mL (Figure 7b). In agreement with the previous results, LP alone did not cause any significant reduction in the CFU number of both *P. aeruginosa* strains (Figure 7a,b).

## 3. Discussion

Lactobacilli are non-pathogenic bacteria closely associated with the human microbiota and commonly used as probiotics. Their probiotic effect relies on their ability to modulate the host immune system, enhance the epithelial barrier function, or fight pathogen colonization via competitive exclusion or antimicrobial molecule production [29]. The employment of antimicrobial probiotics (or their products) to fight infectious diseases is supported by the fact that such health-promoting bacteria exert inhibitory activities even against multi-drug-resistant pathogens; furthermore, it is assumed that the loss of their activity against the targeted pathogens is an unlikely or rare event, in contrast to the frequent development of resistance by human pathogens towards antibiotics commonly used in the clinic [30,31]. Thus, in the era of antimicrobial resistance, the possible use of probiotics as antibacterial agents is an emerging issue with a rapidly expanding field of applications [32]. 

Some species of lactobacilli administered by oropharyngeal application or via orogastric or nasogastric tube have already been tested, with different levels of success, in mechanically ventilated patients to fight *P. aeruginosa* pneumonia [33,34]. Similarly, in some studies, oral administration of probiotics to CF patients reduced the rate of *P. aeruginosa* lung infections mainly through mechanisms ascribable to the gut–lung axis [35]. We hypothesized that the antibacterial effect of probiotics against *P. aeruginosa* could be stronger by administering them directly into the respiratory tract. Therefore, in view of future clinical applications of probiotics via the respiratory route, we seek to investigate the potential of lactobacilli as alternative antimicrobial therapy against *P. aeruginosa* in CF in vitro by evaluating their antimicrobial and antibiofilm effects in an artificial sputum medium, closely resembling the CF lung environment. 

The lactobacilli selected for this study were isolated from commercial products to test strains that were already authorized for human use. Antagonistic activities of probiotic bacteria require a specific capacity to survive and/or to grow in the targeted ecosystem; thus, their ability to survive and replicate in ASM was first evaluated. Among the lactobacilli tested, only LP and LRM showed the ability to grow significantly in ASM, highlighting the importance of testing niche-specific conditions to fully explore the site-specific therapeutic potential of probiotic strains. 

To date, most of the studies on the antibacterial activity of probiotics have focused on bacteria-free supernatants of lactobacilli [36,37,38]. Contrarily, only a few reports have investigated the antibacterial and antibiofilm activity of live lactobacilli against *P. aeruginosa* [23,24,39,40]. Herein, we tested the ability of live LP and LRM to exert a bactericidal effect against *P. aeruginosa* in ASM. LP was found to significantly reduce the viable count of PaCF1 and PaCF4, two *P. aeruginosa* strains isolated from the sputum of chronically infected CF patients. The reduction in *P. aeruginosa* viable count in the presence of LP correlated with a marked lowering of the pH of ASM in the co-cultures, suggesting that at least part of the antibacterial effect could be due to the generation of acidic conditions unfavorable for *P. aeruginosa* survival. Being hetero-fermenters, lactobacilli produce various metabolites, such as acetic, lactic, and propionic acids, that may promote the establishment of an acidic environment [41]. Our findings support and expand previous studies where the supernatants of *Lactiplantibacillus plantarum* (strain WCFS1) and *Lacticaseibacillus rhamnosus* (strain GG) were found to inhibit *P. aeruginosa* in a pH-dependent manner in LB medium; such an effect was abolished if the supernatants of both lactobacilli were buffered to a neutral pH [23]. 

In addition to the acidification of the medium, other factors may contribute to the antibacterial effect of lactobacilli, including the production of antimicrobial molecules such as bacteriocins reported to be active in acidic conditions [42]. *Lactiplantibacillus plantarum* produces bacteriocins named plantaricins; among them, plantaricin F was found to inhibit *P. aeruginosa* ATCC 27853 [43,44,45]. Interestingly, it has been previously reported that *L. plantarum* WCFS1 “senses” the presence of *P. aeruginosa* by the detection of the quorum-sensing molecule *N*-3-Oxododecanoyl Homoserine Lactone (3OC_12_) and responds to the presence of such molecule by upregulating the plantaricin gene [46]. Such upregulation of the plantaricin gene could further contribute to the antibacterial effect of *Lactiplantibacillus plantarum* towards *P. aeruginosa*. 

Bacterial aggregation between cells of the same strain is known as auto-aggregation, while aggregation between different strains is known as co-aggregation [47]. It has been demonstrated that *P. aeruginosa* forms more numerous and larger aggregates in the lungs of CF patients who failed eradication therapy than those who successfully cleared their infection [48]. Indeed, bacterial auto-aggregation is known to be a mechanism of antibiotic resistance; in the lungs of CF patients, the auto-aggregation of *P. aeruginosa* was associated with tolerance to high doses of tobramycin [49].

The ability of lactobacilli to co-aggregate with pathogens is considered part of their probiotic activity and has been reported to depend on the tested strain, the bacterial pathogen, and the incubation conditions [50]. This study observed that LP, but not LRM, co-aggregates with *P. aeruginosa* strains from CF lungs. The ability of LP to co-aggregate with *P. aeruginosa* could therefore interfere with the auto-aggregation of *P. aeruginosa* during lung infections. At the same time, the close vicinity between the two bacterial species in the co-aggregates could facilitate the antibacterial effect of molecules released by lactobacilli (e.g., acids, bacteriocins) against *P. aeruginosa*. The co-aggregation effect of lactobacilli towards *P. aeruginosa* might be due to the synthesis of several aggregation-promoting factors (APFs)—i.e., proteins associated with various functional roles, including self-aggregation, co-aggregation with other commensal or pathogenic bacteria, and maintenance of cell shape [51]. Based on in silico analyses, at least seven APFs of *L. plantarum* WCFS1 contain domains predicted to be involved in binding to mucus [51]. Thus, the capacity of lactobacilli to bind mucus may also hamper the adhesion of *P. aeruginosa* to mucin and, subsequently, the formation of auto-aggregates in the CF lungs.

It is known that the ability to form biofilm plays a pivotal role in CF airway colonization by *P. aeruginosa* [52]. In particular, EPS plays a critical role in pathogenicity, antibiotic tolerance, and long-term infection of *P. aeruginosa*. It shields *P. aeruginosa* from the physical and chemical stresses of the host and antibiotic treatments [51]. In this study, we observed that both LP and LRM caused a reduction in the biomass of preformed biofilm of CF strains of *P. aeruginosa*, but not in the number of biofilm-associated bacteria. This finding suggested the hypothesis that lactobacilli could play a role in reducing the EPS of *P. aeruginosa* biofilms. Differential fluorescence staining of biofilm components (namely bacterial cells and EPS) and analysis by confocal microscopy strongly supported our hypothesis, indicating a marked reduction of EPS in biofilms formed by both PaCF1 (non-mucoid) and PaCF11 (mucoid) strains treated with *L. plantarum*. Several mechanisms may be involved in the *L. plantarum*-mediated reduction of *P. aeruginosa* EPS. For instance, it has been reported that *L. plantarum* produces β-glycosidases capable of cleaving β-polysaccharides, major components of *P. aeruginosa* EPS, that the bacterium probably reuses as substrates in fermentation reactions [53]. Alternatively, given that *P. aeruginosa* itself synthesizes glycoside hydrolases like PelA_h_ and PslG_h_ that are involved in the formation and maintenance of the structure of the biofilm scaffold [54], we speculate that the reduction of EPS in biofilm treated with *L. plantarum* may also be caused by an enhanced expression of *P. aeruginosa* glycoside hydrolase genes induced by *L. plantarum*. Finally, we cannot exclude that *L. plantarum* may act on the QS-system of *P. aeruginosa*, which is also involved in the regulation of extracellular polysaccharides synthesis [55]. Indeed, in a recent paper, it has been demonstrated that *L. plantarum* can affect the expression of QS-associated genes. In particular, *L. plantarum* caused a decrease in the *rlhR* gene expression levels and, vice versa, induced an increase in the *lasI* gene levels [56].

In CF patients with a chronic *P. aeruginosa* infection, the European Cystic Fibrosis Society guidelines recommend a tobramycin-inhaled solution on alternate months for patients with moderate-to-severe lung disease to maintain lung health [57]. CF sputum is known to partially inactivate aminoglycosides like tobramycin due to the high concentrations of divalent cations, low pH, and presence of extracellular DNA and mucin that may sequester antibiotic molecules [58]. In vitro investigations have suggested that bioactive tobramycin concentrations may be 10–25-fold lower than the total drug concentration of the antibiotic in CF sputum [59]. In addition, the penetration of tobramycin through *P. aeruginosa* biofilms is likely hampered by the high concentrations of extracellular DNA and polysaccharides (alginate, Psl, and Pel) in the biofilm EPS due to electrostatic attraction between the positive charges of tobramycin and the negatively charged DNA and biofilm polysaccharides [60,61,62].

Interestingly, in this study, LP was found to increase the activity of tobramycin against the pre-formed biofilms of both PaCF1 and PaCF11 strains; this is compatible with our observation of LP-mediated reduction of biofilm EPS. The reduction in EPS amount in the presence of LP could indeed favor the diffusion of tobramycin through biofilm layers and, therefore, the antibiotic interaction with biofilm cells leading to an enhancement of its killing activity. Despite the synergism between LP and tobramycin, an eradicating effect was not observed in the in vitro model, suggesting that a tolerant bulk of bacteria with persister-like features may survive tobramycin treatment. Enzymatic disruption of exopolysaccharides in *P. aeruginosa* biofilm has been previously proposed as a promising therapeutic approach for treating biofilm infections. In a recent study, the disruption of the extracellular polymeric network of *P. aeruginosa* biofilms by alginate lyase from a marine *Pseudoalteromonas* bacterium was reported to enhance the bactericidal properties of tobramycin and ciprofloxacin against preformed biofilm [63]. Nevertheless, a critical factor of such an approach could be the presence of inhibitors of the enzyme activity in the lung fluid of chronically infected CF patients. In this regard, another study demonstrated that when the biofilm of a mucoid strain of *P. aeruginosa* was exposed to a commercial alginate lyase, the enzyme did not significantly reduce the biofilm biomass, and thus, it did not enhance the antibiofilm activity of antibiotics [64]. The low activity of the commercial alginate lyase was due to the inhibitory effects of calcium and zinc cations at concentrations found in the lung of CF patients, indicating that testing the effectiveness of new antimicrobial strategies must consider the local conditions found in the infectious site. In the present study, the tobramycin-enhancing effect exerted by live *L. plantarum* was observed in the presence of ASM, opening new possibilities of probiotic + antibiotic combinations for treating chronic *P. aeruginosa* lung infections in CF patients.

## 4. Material and Methods

### 4.1. Bacterial Strains and Growth Conditions

The *P. aeruginosa* strains used in the study (PaCF1, PaCF4, and PaCF11; Table S2) were isolated from sputum samples of chronically infected CF patients. They are part of a collection of strains from the Microbiology Laboratories at the University of Pisa and Chieti-Pescara. Seven species/strains of lactobacilli isolated from commercial products in Italy were initially used to test their ability to grow in ASM (Table S1). Identification of all the strains at the species level were performed by MALDI-TOF (Bruker Daltonics, Bremen, Germany). For the preparation of stock cultures, bacterial strains were grown in Luria Bertani broth (*P. aeruginosa*) (LB, Oxoid, Basingstoke, Hampshire, UK) or in De Man–Rogosa–Sharpe broth (lactobacilli) (MRSB, Oxoid, Basingstoke, Hampshire, UK) until the late-log phase, subdivided in aliquots, and kept frozen at −80 °C until use. For CFU count, *P. aeruginosa* was grown on Tryptone Soy Agar (TSA) or MacConkey agar while lactobacilli on the De Man–Rogosa–Sharpe agar (MRSA, Oxoid, Basingstoke, Hampshire, UK).

### 4.2. Determination of Minimum Inhibitory Concentrations (MICs)

The susceptibility of *P. aeruginosa* strains to tobramycin was assessed by the standard broth microdilution method according to the European Committee on Antimicrobial Susceptibility Testing (EUCAST http://www.eucast.org/clinical_breakpoints, accessed on 1 January 2023). Briefly, *P. aeruginosa* was grown in Muller–Hinton broth (MHB, Oxoid, Basingstoke, Hampshire, UK) until the exponential growth phase and diluted in the same medium to reach a final density of 5 × 10^6^ CFU/mL. A total of 10 μL from the bacterial suspension was added to 90 μL of MHB in a 96-well plate in the absence (viability control) or in the presence of tobramycin (Merck, Milan, Italy) at different concentrations. MIC values were defined as the lowest concentration of tobramycin resulting in the inhibition of visible growth after 24 h of incubation at 37 °C. The same procedure with some modifications was used to test the sensitivity of lactobacilli against tobramycin. MICs were evaluated in lactobacilli susceptibility test medium, a mixed formulation containing 90% Iso-Sensitest broth (Oxoid, Basingstoke, Hampshire, UK), and 10% MRSB supplemented with 0.05% (*wt*/*vol*) L-cysteine (Merck, Milan, Italy), as described in the ISO 10932 document [65] and recommended by the EFSA [66].

### 4.3. Preparation of Artificial Sputum Medium (ASM)

For ASM preparation, the following amounts of the different components were dissolved into 20 mL of sterile milliQ water: 100 mg mucin from pig stomach (Merck, Milan, Italy); 80 mg unsheared salmon sperm DNA (Merck, Milan, Italy); 100 mg NaCl; 44 mg KCl; 0.1 mL egg yolk emulsion (Merck, Milan, Italy); 0.12 mg diethylenetriaminepentaacetic acid (Merck, Milan, Italy); 100 mg casamino acids (Gibco); and 11.4 mg glucose (Merck, Milan, Italy). The pH of the solution was adjusted at 6.8 with HCl [25]. Each experiment was carried out using ASM freshly prepared or stored at 4 °C for up to one week.

### 4.4. Evaluation of Lactobacilli Growth in ASM and Their Antibacterial Activity

Overnight cultures of each strain/species of lactobacilli were diluted in ASM to obtain a suspension of 10^5^ CFU/mL, and a volume of 200 μL/well was seeded in 96-well plates (Corning Costar, Lowell, MA, USA). After 24 h of incubation at 37 °C under static and microaerophilic conditions, serial dilutions of the bacterial suspensions were plated on MRSA, and the number of CFU/mL was compared with that of the initial inoculum.

To test the antibacterial activity of LP and LRM against *P. aeruginosa* PaCF1 and PaCF4 in ASM, a suspension of 2 × 10^5^ CFU/mL of lactobacilli was grown for 9 h in 100 μL of ASM, before adding 100 μL of ASM containing 2 × 10^3^ CFU/mL of PaCF1 or PaCF4. The incubation was prolonged for an additional 13 h. Each bacterial strain was also incubated alone in ASM. The 96-well plates were incubated at 37 °C in static and microaerophilic conditions. After 22 h of incubation, dilutions of suspensions were plated on MRSA for lactobacilli enumeration and on MacConkey agar for *P. aeruginosa* enumeration.

### 4.5. Coaggregation Assay

The coaggregation assay was performed as previously described [67]. Briefly, lactobacilli and *P. aeruginosa* were grown overnight in MRSB and LB, respectively, washed twice, and resuspended in phosphate-buffered saline (PBS, Euroclone SpA, Pero, Milan, Italy). Optical density (OD_600_) was adjusted to 0.2 corresponding to a viable count of approximately 10^8^ CFU/mL. Equal volumes of cells (7.5 mL) of the different probiotic and pathogenic strains were mixed, vortexed for 10 s, and incubated at room temperature in static conditions. Coaggregation, evidenced as a decrease of the absorbance in the upper layers of the tube, was determined by monitoring the OD_600 nm_ during 5 h of incubation. The percentage of coaggregation was calculated using the equation:

$$Coaggregation\;\left(\%\right)=\frac{\frac{Ax+Ay}{2}-A\left(x+y\right)}{\frac{Ax+Ay}{2}}\cdot 100$$

where *Ax* and *Ay* represent the absorbance of the separate bacterial suspensions in control tubes, and *A*(*x* + *y*) represents the absorbance of the mixed bacterial suspension at the different times tested.

### 4.6. Biofilm Inhibition Assay

Bacterial strains were grown overnight at 37 °C in LB (PaCF1 and PaCF4) or MRSB (LP and LRM). Following incubation, *P. aeruginosa* suspensions were diluted 1:50 in ASM and mixed with each strain of lactobacilli, previously diluted in ASM at different concentrations (10^6^, 10^7^, 10^8^ CFU/mL), in a final volume of 100 μL. Bacterial suspensions were seeded into wells of a flat-bottom polystyrene 96-well microtiter plate. Wells containing single bacterial strains were prepared as controls. Plates were incubated for 24 h in static conditions at 37 °C to let the biofilms grow. Following incubation, wells were gently washed thrice with PBS to remove non-biofilm-embedded bacteria. In a set of experiments, biofilms were quantified by staining with 0.5% crystal violet (CV), according to previously described procedures [68]. In another set of experiments, the CFU count of biofilm-associated bacteria was performed. To this aim, the surface of each well was scraped using a sterile tip to detach biofilms that were resuspended in 1 mL PBS. To disaggregate the biofilm cells, bacterial suspensions were vortexed for 30 s, sonicated for 30 s in a water bath sonicator (Ultrasonic cleaner, VWR), and vortexed for a further 30 s. Serially diluted bacterial suspensions were plated on TSA agar plates and incubated for 24–48 h for CFU enumeration.

### 4.7. Eradication of Preformed Biofilm Assay

Bacterial strains PACF1, PaCF4, and PaCF11 were grown in agitation overnight in LB. Following incubation, each bacterial suspension was diluted 1:50 in ASM and a volume of 100 μL was distributed in a 96-well microtiter plate and incubated at 37 °C for 24 h, to let the biofilms grow. Following incubation, non-adherent bacteria were removed by two washes with PBS and biofilms added with LP or LRM grown overnight in MRSB. They were then diluted in ASM at different concentrations (10^6^, 10^7^, 10^8^ CFU/mL). Control samples were incubated in the presence of ASM only. After a further incubation of 24 h, the biofilm formation was evaluated by CV staining or by enumerating the number of biofilm-associated viable bacteria (Section 4.6).

In a set of experiments, following the treatment with lactobacilli, biofilms were further incubated for 24 h with tobramycin at concentrations ranging from 8 to 64 μg/mL. Control samples were incubated in the presence of ASM or tobramycin only. After washing with PBS, the evaluation of the number of biofilm-associated viable bacteria was performed.

### 4.8. Confocal Laser Scanning Microscopy Analysis of P. aeruginosa Biofilm after Exposure to L. plantarum

Pre-formed biofilms (24-h-old) of PaCF1 and PaCF11 treated with LP for 24 h (as described in Section 4.7) were labeled with 0.4% PHK26 (Merck, Milan, Italy), an orange fluorescent lipophilic dye, according to the manufacturer’s instructions. After washings to remove unbound PHK26, biofilms were incubated in 200 μL of Calcofluor white (Merck, Milan, Italy), at a concentration of 500 μg/mL in KOH 10% for 1 min, to stain β-polysaccharides. After further washes with PBS to remove the excess of calcofluor white, biofilms were kept protected from light until imaging. Biofilms were visualized by using the Operetta CLS High-Content Analysis System (PerkinElmer Inc., Boston, MA, USA), acquiring 29 plane confocal images at 63× magnification for each biofilm. Images were then analyzed by Harmony software (Version 4.9, Perkin Elmer Inc., Boston, MA, USA).

### 4.9. Statistical Analysis

All the experiments were performed at least three times. Statistical analysis was carried out using GraphPad InStat software (GraphPad InStat Software version 3.06, Inc., San Diego, CA, USA). Differences between mean values were evaluated by the Student’s *t*-test or one-way analysis of variance (ANOVA) followed by the Tukey–Kramer posthoc test. A *p*-value of <0.05 was considered significant.

## 5. Conclusions

To the best of our knowledge, in the present study, the potential of live lactobacilli as antibacterial agents was tested for the first time in conditions mimicking the CF lung. We demonstrated that commercial lactobacilli strains differ in their ability to grow/survive in such conditions. Overall LRM and LP showed a good ability to grow in ASM and to contrast *P. aeruginosa* growth in planktonic and/or biofilm mode of growth. Noteworthy, LP was able to interfere with the formation of EPS in *P. aeruginosa* mature biofilms and to markedly enhance the bactericidal effect of tobramycin against the same biofilms in ASM. Overall, our results suggest LP as a promising candidate to address future studies both in in vitro and in vivo models of infection aimed at clarifying whether such probiotics can be used as adjuvant for the therapy of lung infections by *P. aeruginosa* in CF patients.

## Data Availability

Data are available upon request to the corresponding authors.

## Supplementary Materials

The following supporting information can be downloaded at: https://www.mdpi.com/article/10.3390/antibiotics12071158/s1, Table S1: Lactobacilli species/strains used in the study; Table S2: Characterization of *P. aeruginosa* strains isolated from CF patients; Figure S1: Ability of lactobacilli to grow/survive in artificial sputum medium (ASM) without glucose; Figure S2: pH values of PaCF1 and PaCF4 liquid cultures grown alone and co-cultured with LP and LRM; Figure S3: Effects of LP and LRM on *P. aeruginosa* biofilm evaluated by CFU count.

## References

[1] Turcios N.L. (2020). Cystic Fibrosis Lung Disease: An Overview. Respir. Care.

[2] Hall-Stoodley L., Costerton J.W., Stoodley P. (2004). Bacterial biofilms: From the Natural environment to infectious diseases. Nat. Rev. Genet..

[3] Willner D.L., Haynes M.R., Furlan M., Schmieder R., Lim Y.W., Rainey P.B., Rohwer F., Conrad D. (2012). Spatial distribution of microbial communities in the cystic fibrosis lung. ISME J..

[4] Limoli D.H., Whitfield G.B., Kitao T., Ivey M.L., Davis M.R., Grahl N., Hogan D.A., Rahme L.G., Howell P.L., O’Toole G.A. (2017). *Pseudomonas aeruginosa* Alginate Overproduction Promotes Coexistence with *Staphylococcus aureus* in a Model of Cystic Fibrosis Respiratory Infection. mBio.

[5] Armbruster C.R., Wolter D.J., Mishra M., Hayden H.S., Radey M.C., Merrihew G., MacCoss M.J., Burns J., Wozniak D.J., Parsek M.R. (2016). *Staphylococcus aureus* Protein A Mediates Interspecies Interactions at the Cell Surface of *Pseudomonas aruginosa*. mBio.

[6] Malhotra S., Hayes D., Wozniak D.J. (2019). Cystic Fibrosis and *Pseudomonas aeruginosa*: The Host-Microbe Interface. Clin. Microbiol. Rev..

[7] Yin R., Cheng J., Wang J., Li P., Lin J. (2022). Treatment of *Pseudomonas aeruginosa* Infectious Biofilms: Challenges and Strategies. Front. Microbiol..

[8] Harvey C., Weldon S., Elborn S., Downey D.G., Taggart C. (2022). The Effect of CFTR Modulators on Airway Infection in Cystic Fibrosis. Int. J. Mol. Sci..

[9] Thornton C.S., Acosta N., Surette M.G., Parkins M.D. (2022). Exploring the Cystic Fibrosis Lung Microbiome: Making the Most of a Sticky Situation. J. Pediatr. Infect. Dis. Soc..

[10] Elborn J.S., Blasi F., Burgel P.-R., Peckham D. (2023). Role of Inhaled Antibiotics in the Era of Highly Effective CFTR Modulators. Eur. Respir. Rev..

[11] Einarsson G.G., Ronan N.J., Mooney D., McGettigan C., Mullane D., NiChroinin M., Shanahan F., Murphy D.M., McCarthy M., McCarthy Y. (2021). Extended-Culture and Culture-Independent Molecular Analysis of the Airway Microbiota in Cystic Fibrosis Following CFTR Modulation with Ivacaftor. J. Cyst. Fibros..

[12] Hisert K.B., Heltshe S.L., Pope C., Jorth P., Wu X., Edwards R.M., Radey M., Accurso F.J., Wolter D.J., Cooke G. (2017). Restoring Cystic Fibrosis Transmembrane Conductance Regulator Function Reduces Airway Bacteria and Inflammation in People with Cystic Fibrosis and Chronic Lung Infections. Am. J. Respir. Crit. Care Med..

[13] Batoni G., Maisetta G., Kaya E., Esin S. (2022). Lung-Directed Bacteriotherapy in Cystic Fibrosis: Could It Be an Option?. Antibiotics.

[14] Das T.K., Pradhan S., Chakrabarti S., Mondal K.C., Ghosh K. (2022). Current Status of Probiotic and Related Health Benefits. Appl. Food Res..

[15] Milner E., Stevens B., An M., Lam V., Ainsworth M., Dihle P., Stearns J., Dombrowski A., Rego D., Segars K. (2021). Utilizing Probiotics for the Prevention and Treatment of Gastrointestinal Diseases. Front. Microbiol..

[16] Weiss B., Bujanover Y., Yahav Y., Vilozni D., Fireman E., Efrati O. (2010). Probiotic Supplementation Affects Pulmonary Exacerbations in Patients with Cystic Fibrosis: A Pilot Study. Pediatr. Pulmonol..

[17] Bruzzese E., Raia V., Spagnuolo M.I., Volpicelli M., De Marco G., Maiuri L., Guarino A. (2007). Effect of Lactobacillus GG Supplementation on Pulmonary Exacerbations in Patients with Cystic Fibrosis: A Pilot Study. Clin. Nutr..

[18] Jafari S.-A., Mehdizadeh-Hakkak A., Kianifar H.-R., Hebrani P., Ahanchian H., Abbasnejad E. (2013). Effects of Probiotics on Quality of Life in Children with Cystic Fibrosis; a Randomized Controlled Trial. Iran. J. Pediatr..

[19] Neri L.D.C.L., Taminato M., da Silva L.V.R.F. (2019). Systematic Review of Probiotics for Cystic Fibrosis Patients: Moving Forward. J. Pediatr. Gastroenterol. Nutr..

[20] Fangous M.S., Alexandre Y., Hymery N., Gouriou S., Arzur D., Blay G.L., Berre R.L. (2019). Lactobacilli Intra-Tracheal Administration Protects from *Pseudomonas aeruginosa* Pulmonary Infection in Mice—A Proof of Concept. Benef. Microbes.

[21] Fangous M.-S., Gosset P., Galakhoff N., Gouriou S., Guilloux C.-A., Payan C., Vallet S., Héry-Arnaud G., Le Berre R. (2021). Priming with Intranasal Lactobacilli Prevents *Pseudomonas aeruginosa* Acute Pneumonia in Mice. BMC Microbiol..

[22] Fangous M.-S., Lazzouni I., Alexandre Y., Gouriou S., Boisramé S., Vallet S., Le Bihan J., Ramel S., Héry-Arnaud G., Le Berre R. (2018). Prevalence and Dynamics of *Lactobacillus* sp. in the Lower Respiratory Tract of Patients with Cystic Fibrosis. Res. Microbiol..

[23] Jamalifar H., Rahimi H., Samadi N., Shahverdi A., Sharifian Z., Hosseini F., Eslahi H., Fazeli M. (2011). Antimicrobial Activity of Different *Lactobacillus* species against Multi- Drug Resistant Clinical Isolates of *Pseudomonas aeruginosa*. Iran. J. Microbiol..

[24] Shokri D., Khorasgani M.R., Mohkam M., Fatemi S.M., Ghasemi Y., Taheri-Kafrani A. (2018). The Inhibition Effect of Lactobacilli against Growth and Biofilm Formation of *Pseudomonas aeruginosa*. Probiotics Antimicrob. Proteins.

[25] Sriramulu D.D., Lünsdorf H., Lam J.S., Römling U. (2005). Microcolony Formation: A Novel Biofilm Model of *Pseudomonas aeruginosa* for the Cystic Fibrosis Lung. J. Med. Microbiol..

[26] Palmer K.L., Aye L.M., Whiteley M. (2007). Nutritional Cues Control *Pseudomonas aeruginosa* Multicellular Behavior in Cystic Fibrosis Sputum. J. Bacteriol..

[27] Younes J.A., van der Mei H.C., van den Heuvel E., Busscher H.J., Reid G. (2012). Adhesion Forces and Coaggregation between Vaginal Staphylococci and Lactobacilli. PLoS ONE.

[28] Baird F.J., Wadsworth M.P., Hill J.E. (2012). Evaluation and Optimization of Multiple Fluorophore Analysis of a *Pseudomonas aeruginosa* Biofilm. J. Microbiol. Methods.

[29] Raheem A., Liang L., Zhang G., Cui S. (2021). Modulatory Effects of Probiotics During Pathogenic Infections with Emphasis on Immune Regulation. Front. Immunol..

[30] Simons A., Alhanout K., Duval R.E. (2020). Bacteriocins, Antimicrobial Peptides from Bacterial Origin: Overview of Their Biology and Their Impact against Multidrug-Resistant Bacteria. Microorganisms.

[31] Todorov S.D., de Melo Franco B.D.G., Tagg J.R. (2019). Bacteriocins of Gram-Positive Bacteria Having Activity Spectra Extending beyond Closely-Related Species. Benef. Microbes.

[32] Silva D.R., Sardi J.d.C.O., Pitangui N.d.S., Roque S.M., Silva A.C.B.d., Rosalen P.L. (2020). Probiotics as an Alternative Antimicrobial Therapy: Current Reality and Future Directions. J. Funct. Foods.

[33] Siempos I.I., Ntaidou T.K., Falagas M.E. (2010). Impact of the Administration of Probiotics on the Incidence of Ventilator-Associated Pneumonia: A Meta-Analysis of Randomized Controlled Trials. Crit. Care Med..

[34] Hao Q., Lu Z., Dong B.R., Huang C.Q., Wu T. (2011). Probiotics for Preventing Acute Upper Respiratory Tract Infections. Cochrane Database Syst. Rev..

[35] Esposito S., Testa I., Mariotti Zani E., Cunico D., Torelli L., Grandinetti R., Fainardi V., Pisi G., Principi N. (2022). Probiotics Administration in Cystic Fibrosis: What Is the Evidence?. Nutrients.

[36] Jeyanathan A., Ramalhete R., Blunn G., Gibbs H., Pumilia C.A., Meckmongkol T., Lovejoy J., Coathup M.J. (2021). *Lactobacillus* Cell-Free Supernatant as a Novel Bioagent and Biosurfactant against *Pseudomonas aeruginosa* in the Prevention and Treatment of Orthopedic Implant Infection. J. Biomed. Mater. Res. B Appl. Biomater..

[37] El-Mokhtar M.A., Hassanein K.M., Ahmed A.S., Gad G.F., Amin M.M., Hassanein O.F. (2020). Antagonistic Activities of Cell-Free Supernatants of Lactobacilli Against Extended-Spectrum β-Lactamase Producing *Klebsiella pneumoniae* and *Pseudomonas aeruginosa*. Infect. Drug Resist..

[38] Chappell T.C., Nair N.U. (2020). Engineered Lactobacilli Display Anti-Biofilm and Growth Suppressing Activities against *Pseudomonas aeruginosa*. NPJ Biofilms Microbiomes.

[39] Abootaleb M., Mohammadi Bandari N., Arbab Soleimani N. (2022). Interference of *Lactiplantibacillus plantarum* with *Pseudomonas aeruginosa* on the Infected Burns in Wistar Rats. J. Burn. Care Res..

[40] Khalfallah G., Gartzen R., Möller M., Heine E., Lütticken R. (2021). A New Approach to Harness Probiotics against Common Bacterial Skin Pathogens: Towards Living Antimicrobials. Probiotics Antimicrob. Proteins.

[41] Wang Y., Wu J., Lv M., Shao Z., Hungwe M., Wang J., Bai X., Xie J., Wang Y., Geng W. (2021). Metabolism Characteristics of Lactic Acid Bacteria and the Expanding Applications in Food Industry. Front. Bioeng. Biotechnol..

[42] Bhattacharya D., Nanda P.K., Pateiro M., Lorenzo J.M., Dhar P., Das A.K. (2022). Lactic Acid Bacteria and Bacteriocins: Novel Biotechnological Approach for Biopreservation of Meat and Meat Products. Microorganisms.

[43] van den Nieuwboer M., van Hemert S., Claassen E., de Vos W.M. (2016). *Lactobacillus plantarum* WCFS1 and Its Host Interaction: A Dozen Years after the Genome. Microb. Biotechnol..

[44] Choi S., Baek M.-G., Chung M.-J., Lim S., Yi H. (2021). Distribution of Bacteriocin Genes in the Lineages of *Lactiplantibacillus plantarum*. Sci. Rep..

[45] Fricourt B.V., Barefoot S.F., Testin R.F., Hayasaka S.S. (1994). Detection and Activity of Plantaricin F an Antibacterial Substance from *Lactobacillus plantarum* BF001 Isolated from Processed Channel Catfish. J. Food Prot..

[46] Spangler J.R., Dean S.N., Leary D.H., Walper S.A. (2019). Response of *Lactobacillus plantarum* WCFS1 to the Gram-Negative Pathogen-Associated Quorum Sensing Molecule N-3-Oxododecanoyl Homoserine Lactone. Front. Microbiol..

[47] Vlková E., Rada V., Smehilová M., Killer J. (2008). Auto-Aggregation and Co-Aggregation Ability in Bifidobacteria and Clostridia. Folia Microbiol. (Praha).

[48] Morris A.J., Yau Y.C.W., Park S., Eisha S., McDonald N., Parsek M.R., Howell P.L., Hoffman L.R., Nguyen D., DiGiandomenico A. (2022). *Pseudomonas aeruginosa* Aggregation and Psl Expression in Sputum Is Associated with Antibiotic Eradication Failure in Children with Cystic Fibrosis. Sci. Rep..

[49] Morris A.J., Jackson L., Cw Yau Y., Reichhardt C., Beaudoin T., Uwumarenogie S., Guttman K.M., Lynne Howell P., Parsek M.R., Hoffman L.R. (2021). The Role of Psl in the Failure to Eradicate *Pseudomonas aeruginosa* Biofilms in Children with Cystic Fibrosis. NPJ Biofilms Microbiomes.

[50] Collado M.C., Meriluoto J., Salminen S. (2008). Adhesion and Aggregation Properties of Probiotic and Pathogen Strains. Eur. Food Res. Technol..

[51] Boekhorst J., Helmer Q., Kleerebezem M., Siezen R.J. (2006). Comparative Analysis of Proteins with a Mucus-Binding Domain Found Exclusively in Lactic Acid Bacteria. Microbiology.

[52] Guillaume O., Butnarasu C., Visentin S., Reimhult E. (2022). Interplay between Biofilm Microenvironment and Pathogenicity of *Pseudomonas aeruginosa* in Cystic Fibrosis Lung Chronic Infection. Biofilm.

[53] Lee N.-K., Paik H.-D. (2017). Bioconversion Using Lactic Acid Bacteria: Ginsenosides, GABA, and Phenolic Compounds. J. Microbiol. Biotechnol..

[54] Baker P., Hill P.J., Snarr B.D., Alnabelseya N., Pestrak M.J., Lee M.J., Jennings L.K., Tam J., Melnyk R.A., Parsek M.R. (2016). Exopolysaccharide Biosynthetic Glycoside Hydrolases Can Be Utilized to Disrupt and Prevent *Pseudomonas aeruginosa* Biofilms. Sci. Adv..

[55] Ueda A., Wood T.K. (2009). Connecting Quorum Sensing, c-Di-GMP, Pel Polysaccharide, and Biofilm Formation in *Pseudomonas aeruginosa* through Tyrosine Phosphatase TpbA (PA3885). PLoS Pathog..

[56] Li M., Xiao H., Su Y., Cheng D., Jia Y., Li Y., Yin Q., Gao J., Tang Y., Bai Q. (2023). Synergistic Inhibitory Effect of Honey and *Lactobacillus plantarum* on Pathogenic Bacteria and Their Promotion of Healing in Infected Wounds. Pathogens.

[57] Castellani C., Duff A.J.A., Bell S.C., Heijerman H.G.M., Munck A., Ratjen F., Sermet-Gaudelus I., Southern K.W., Barben J., Flume P.A. (2018). ECFS Best Practice Guidelines: The 2018 Revision. J. Cyst. Fibros..

[58] Hunt B.E., Weber A., Berger A., Ramsey B., Smith A.L. (1995). Macromolecular Mechanisms of Sputum Inhibition of Tobramycin Activity. Antimicrob. Agents Chemother..

[59] Mendelman P.M., Smith A.L., Levy J., Weber A., Ramsey B., Davis R.L. (1985). Aminoglycoside Penetration, Inactivation, and Efficacy in Cystic Fibrosis Sputum. Am. Rev. Respir. Dis..

[60] Chiang W.-C., Nilsson M., Jensen P.Ø., Høiby N., Nielsen T.E., Givskov M., Tolker-Nielsen T. (2013). Extracellular DNA Shields against Aminoglycosides in *Pseudomonas aeruginosa* Biofilms. Antimicrob. Agents Chemother..

[61] Nichols W.W., Dorrington S.M., Slack M.P., Walmsley H.L. (1988). Inhibition of Tobramycin Diffusion by Binding to Alginate. Antimicrob. Agents Chemother..

[62] Colvin K.M., Gordon V.D., Murakami K., Borlee B.R., Wozniak D.J., Wong G.C.L., Parsek M.R. (2011). The Pel Polysaccharide Can Serve a Structural and Protective Role in the Biofilm Matrix of *Pseudomonas aeruginosa*. PLoS Pathog..

[63] Daboor S.M., Rohde J.R., Cheng Z. (2021). Disruption of the Extracellular Polymeric Network of *Pseudomonas aeruginosa* Biofilms by Alginate Lyase Enhances Pathogen Eradication by Antibiotics. J. Cyst. Fibros..

[64] Germoni L.A.P., Bremer P.J., Lamont I.L. (2016). The Effect of Alginate Lyase on the Gentamicin Resistance of *Pseudomonas aeruginosa* in Mucoid Biofilms. J. Appl. Microbiol..

[65] (2010). Determination of the Minimal Inhibitory Concentration (MIC) of Antibiotics Applicable to Bifidobacteria and Non-Enterococcal Lactic Acid Bacteria (LAB)—Milk and Milk Products.

[66] EFSA Panel on Additives and Products or Substances used in Animal Feed (FEEDAP) (2012). Guidance on the Assessment of Bacterial Susceptibility to Antimicrobials of Human and Veterinary Importance. EFSA J..

[67] Collado M.C., Isolauri E., Salminen S. (2008). Specific Probiotic Strains and Their Combinations Counteract Adhesion of Enterobacter Sakazakii to Intestinal Mucus. FEMS Microbiol. Lett..

[68] Maisetta G., Batoni G., Caboni P., Esin S., Rinaldi A.C., Zucca P. (2019). Tannin Profile, Antioxidant Properties, and Antimicrobial Activity of Extracts from Two Mediterranean Species of Parasitic Plant Cytinus. BMC Complement. Altern. Med..

