# Supplementary material for: Antibacterial and Antibiofilm Effects of Lactobacilli Strains against Clinical Isolates of Pseudomonas aeruginosa under Conditions Relevant to Cystic Fibrosis
Source: Antibiotics (Basel). 2023 Jul 7;12(7):1158. doi: 10.3390/antibiotics12071158 (PMC10376640; doi:10.3390/antibiotics12071158)
Supplement: Supplementary file 1 [file antibiotics-12-01158-s001.zip › antibiotics-2449888-supplementary.pdf]

Table S1. Species/strains of lactobacilli used in the study

Strain Code	Species	Commercial product	Strains declared by the manufacturer
LR ATCC 7469	*Lacticaseibacillus rhamnosus*	//	ATCC 7469
LRM	*Lacticaseibacillus rhamnosus*	Microbiosys	Rosell/GG
LRD	*Lacticaseibacillus rhamnosus*	Dicoflor	GG
LP	*Lactiplantibacillus plantarum*	Biotics G	WCFS1
LF	*Limosilactobacillus fermentum*	Urotab	DSM 25176
LPA	*Lacticaseibacillus paracasei*	Biotics G	W20
LA	*Lactobacillus acidophilus*	Nature's Bounty	LA-14

Table S2. Characterization of *P. aeruginosa* strains isolated from CF patients

Strain code	Sex	Age	Disease stage	Mucoid phenotype (Yes/Not)
PaCF1	F	18	Chronic	N
PaCF4	F	18	Chronic	Y
PaCF11	M	20	Chronic	Y

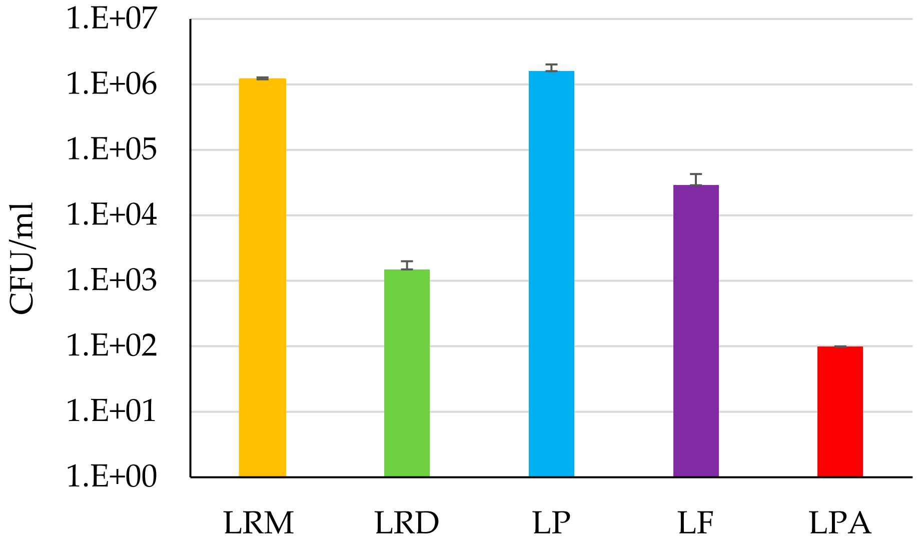

Figure S1. Ability of lactobacilli to grow/survive in artificial sputum medium (ASM) without glucose, starting from an inoculum of 10^5 CFU/ml. The figure reports the CFU number of the different lactobacilli strains after 24 h of incubation in ASM. Results are shown as mean ± standard error of the mean values (n= 3). LRM and LP showed ability to grow in ASM without glucose although at a lower extent as compared to the presence of glucose (1 Log increase *versus* approximately 3 Logs increase, Figure 1). In contrast, the viable number of LRD, LF and LPA decreased in such conditions.

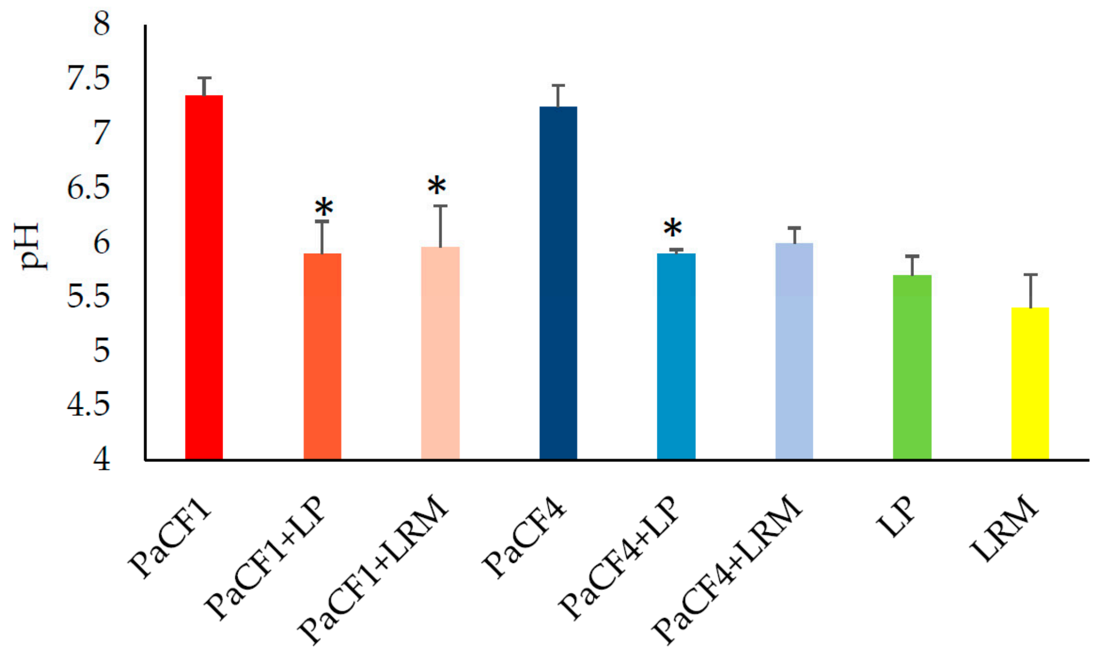

Figure S2. pH values measured by pH-indicator strips in liquid cultures of PaCF1 and PaCF4 alone, and co-cultured with LP and LRM. Lactobacilli were grown in ASM for 9 h before adding *P. aeruginosa*, and the incubation of both bacterial strains was prolonged for an additional 13 h. Results are shown as mean ± error standard of the mean (n= 7). Statistical significance was evaluated by ANOVA followed by Tukey-Kramer post-hoc test. **p*<0.05.

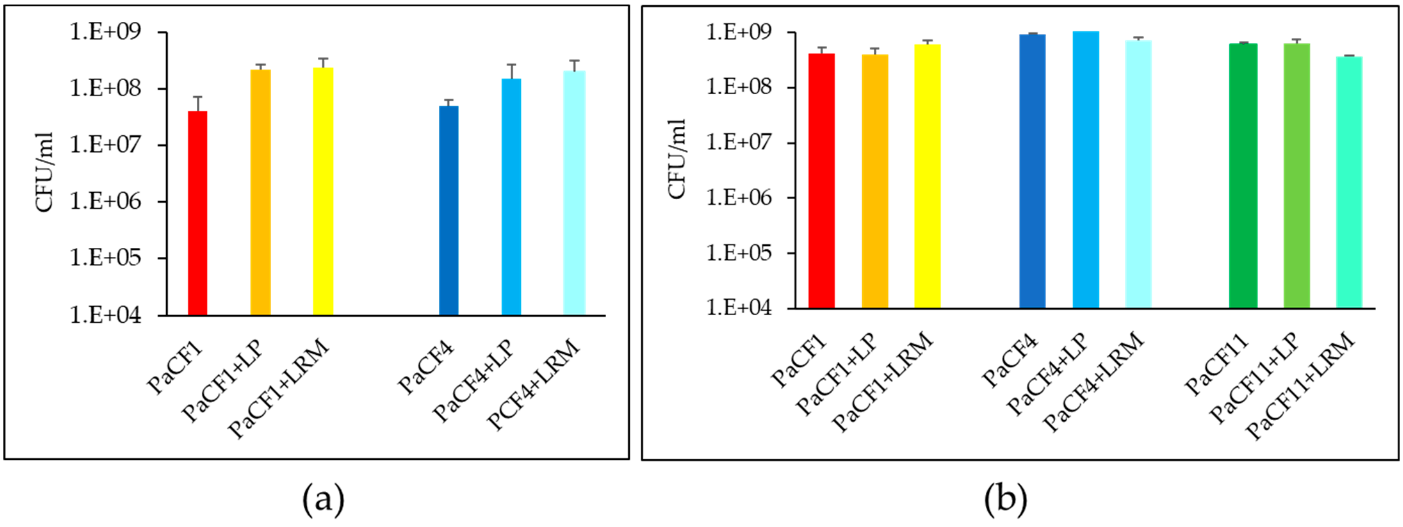

Figure S3. Effects of LP and LRM (tested at 10^8 CFU/mL) on the formation of biofilm by PaCF1 and PaCF4 (a) and on preformed biofilm of PaCF1, PaCF4 and PaCF11 (b). CFU counts of biofilm-associated *P. aeruginosa* were assessed at the end of the incubation period following the washing of the unattached bacteria. Results are shown as mean ± standard error of the mean (n= 3).
